# Three-dimensional automated measurements of femoral angles for the preoperative planning in dogs with patellar luxation

**DOI:** 10.3389/fvets.2024.1456508

**Published:** 2024-11-13

**Authors:** Federico Longo, Sebastian Knell, Tommaso Nicetto, Gianpaolo Savio, Antonio Pozzi, Maurizio Isola

**Affiliations:** ^1^Department of Small Animal Surgery, University of Zurich, Zürich, Switzerland; ^2^Pedrani Veterinary Clinic, Zugliano, Italy; ^3^Department of Civil, Environmental and Architectural Engineering, University of Padua, Padua, Italy; ^4^Department of Small Animal Surgery, University of Padua, Padua, Italy

**Keywords:** 3D computation, femur, dog, patellar luxation, limb deformities

## Abstract

**Objectives:**

To report the surgical outcomes of treating patellar luxation (PL) in dogs with surgical planning based on three-dimensional (3D) automated measurement of femoral angles.

**Study design:**

Multicenter retrospective study.

**Methods:**

Forty-one dogs with PL underwent preoperative computed tomography (CT). Three-dimensional femur models were exported as stereolithographic files, and imported into computer-aided design (CAD) software where 3D measurements were performed. The anatomical laterodistal femoral (aLDFA), femoral neck (FNA), and femoral torsion (FTA) angles were recorded. Surgical records, complications, radiographic femoral postoperative alignment, preoperative and postoperative lameness evaluation, and patellar position were reviewed. The success of the surgical outcome was based on the presence of normal patellar tracking at the last clinical recheck.

**Results:**

Forty-seven limbs were included; 46% of the cases (22/47) were affected by grade 3 PL. Mean (±SD) 3D aLDFA, FNA, and FTA measurements were 101.4° (±3.6), 132.5° (±2.6), and 17.6° (±4.3) in dogs with medial patellar luxation (MPL) and 89.3° (±7.6), 134.8° (±2.9), 36.9° (±5.3) with lateral patellar luxation (LPL), respectively. Based on the 3D preoperative planning, corrective osteotomies were performed in 34 of 47 cases. The mean radiographic follow-up was 4.7 months. At the final follow-up, PL was successfully treated in 45 of 47 cases. Patella reluxated in five cases. In three of five cases, the 3D automated plan was not followed by the surgeon.

**Discussion:**

Surgical treatment of PL based on 3D femoral measurements successfully corrected PL in 45 of 47 cases (96%). This is the first study reporting the use of 3D automated femoral angle measurement in clinical cases affected by PL.

## Introduction

Patellar luxation (PL) is one of the most frequent diseases of the canine hindlimb (1–3), with a reported prevalence ranging from 1.3 to 9.8% (1, 2). Different factors have been proposed to contribute to the etiopathogenesis of PL including angular deformity of the femur. Among them, femoral deformities are one of the most recurrent (4, 5). The canine femur has a complex three-dimensional (3D) morphology, due to the concurrent presence of varus (6, 7), anteversion of the femoral head and neck (5, 8), and procurvatum (9). This may complicate the manual drawing of axes and the measurement of angles, especially in the case of complex concurrent multiplanar bone deformities with multiple centers of rotation angulations, such as it may occur in dogs affected by grade 3 and grade 4 PL (10–12).

The purpose of a corrective osteotomy is to alter the quadriceps mechanism alignment so that the tendency for patellar luxation is eliminated (13). To achieve this goal, an accurate evaluation of long-bone alignment is imperative (4). As a result, a growing research focus has been recently dedicated to 3D measurements (14–17) and 3D-printed osteotomy guides (18–20). A 3D automated approach that measures femoral angles was described in *ex vivo* canine femurs (15). The proposed technique requires the use of computed tomography (CT) and CAD (computer-aided design) software to perform the measurements. A previous study reported the accuracy of 3D measurements, showing that the 3D automated measurements can be reliably used in normal femurs and also in femurs affected by severe osteoarthritis or in chondrodystrophic femurs (14).

Previous studies have demonstrated that CT is superior to radiography for the accurate and precise measurement of femoral alignment parameters (6–8). Computed tomography allows 3D images to be manipulated and accurate measurements of the bone to be made. However, the angles are measured with two-dimensional (2D) images (8). The main benefit of 3D automated computation relies on measurements that are independent from the bone orientation, specific anatomical landmarks, and bone morphology. As a result, the identification of anatomical landmarks (i.e., femoral neck) is no longer required, and thus, the operator-related measurement variability is minimized (14, 21). To date, there are no studies reporting the use of 3D automated measurements of femoral angles for the treatment of PL in dogs.

The purpose of this study was to report the clinical outcomes of the surgical treatment of PL based on an automated 3D measurement of femoral angles to allow the planning of corrective surgery for PL. We hypothesized that measurements of femoral alignment parameters obtained using 3D aCAD can be used during surgical planning leading to successful surgical resolution of PL in >95% of cases. The success for the correction of PL was deemed according to the postoperative clinical (lameness score, patellar tracking, and complications) and the radiographic (postoperative femoral alignment, patellar position) outcomes. The success of the 3D automated preoperative planning was defined in case of no patellar reluxation in agreement with the surgical correction plan.

## Materials and methods

### Study population and inclusion criteria

Data of dogs that underwent surgical treatment of PL from December 2016 to December 2022 were reviewed in the computerized databases of two academic institutions and two referrals to veterinary practices. The inclusion criteria for the study were (1) the use of an automated 3D computation for measuring the femoral angles, (2) a clinical diagnosis of PL, and (3) a clinical and radiographic follow-up of at least 2 months after surgery. A follow-up of a minimum time of 2 months was chosen based on the guidelines proposed by Cook et al. (22). With regard to femoral and tibial measurements, the values from the contralateral healthy side, if a monolateral PL was observed, or previously reported values were used to define a pathologic alignment (8, 12, 23–25).

### Medical and surgical records

The following data were collected: dog signalment, body weight, preoperative and postoperative orthopedic examination, lameness score, direction and grade of PL (26), pre- and postoperative femoral measurements, surgical techniques, and complications were reviewed. Complications were classified as catastrophic, major, and minor (12, 22).

### Preoperative clinical findings

The presence, onset (acute, subacute, chronic), and duration (intermittent vs. persistent) of hindlimb lameness were evaluated. Lameness was assessed by observing the dog walking and trotting from the front and the side and scored (0–5) using a previously validated scale (22). The presence of additional clinical findings such as pain at palpation of other joints, detection of periarticular fibrosis and crepitus during stifle palpation, and presence of other orthopedic diseases in the same hindlimb such as cranial cruciate ligament rupture (CCLR) were reviewed.

### Computed tomographic examination

**Figure 1:**
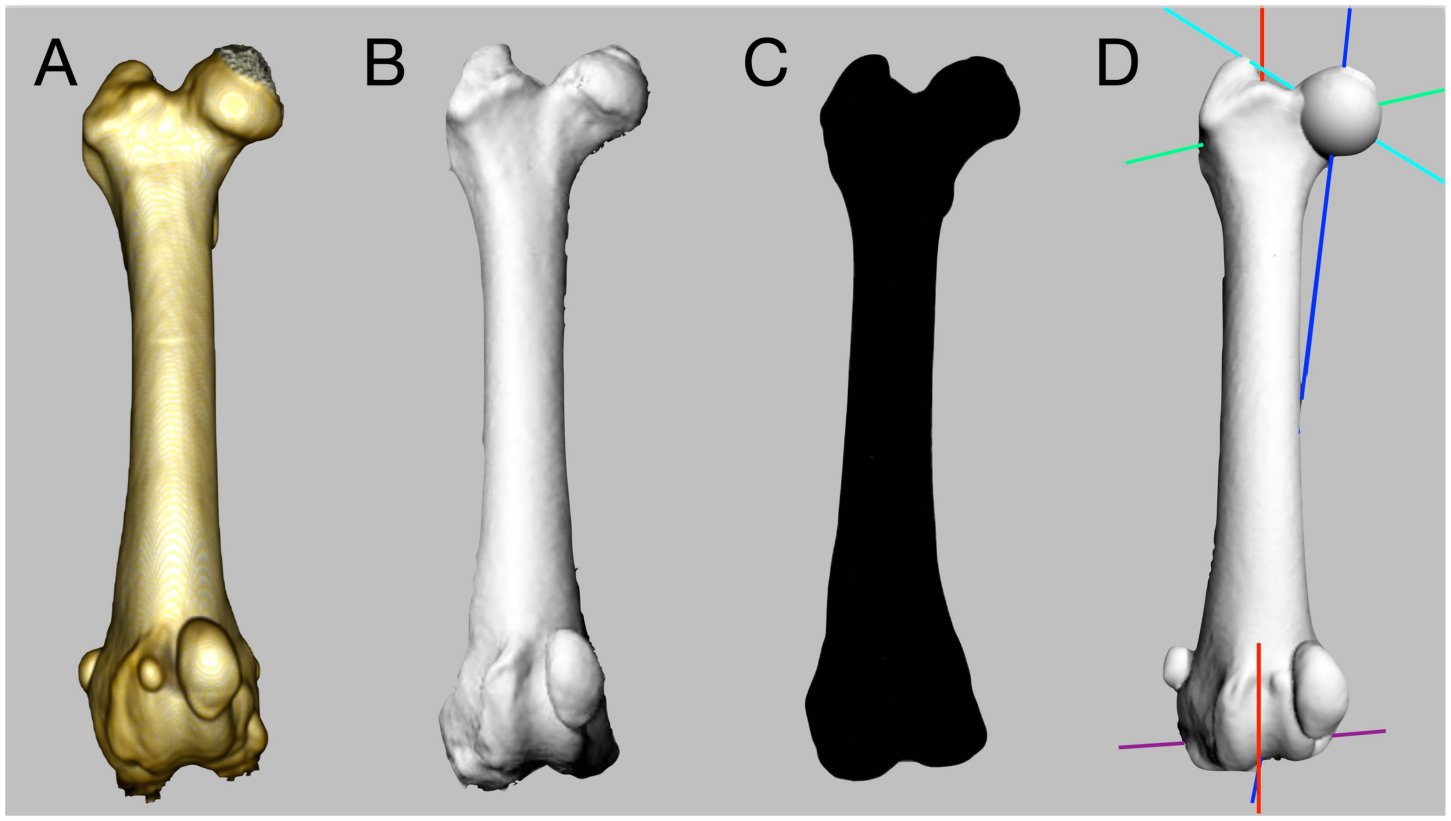
Workflow for the three-dimensional (3D) computational analysis of a canine femur. A computed tomography is performed, and the femur is cropped **(A)**. The isolated femur is segmented and saved as a stereolithographic (STL) file **(B)**. The STL file is imported into computer-aided design software **(C)**. Automated computational analysis is performed **(D)**.

CT scans were performed in a caudocranial direction using a four multidetector row CT scanner (Toshiba Asteion S4, Toshiba Medical Systems Europe), with a slice thickness ranging from 0.8 to 1 mm (reconstruction interval 0.6–0.8 mm). Patients were positioned in dorsal recumbency with the hindlimbs hyperextended and slightly internally rotated. For each hindlimb, the femoral trochlear groove depth and tibial alignment were measured (27, 28). Both femurs were cropped and isolated from the other bony structures (Figure 1A). Segmentation of each femur was performed as previously reported (14, 21). The segmented femur was saved and opened with the 3D surface rendering function (Figure 1B), allowing the 3D femur model reconstruction to be exported as a stereolithographic (STL) file.

### Three-dimensional measurement of femoral angles

The 3D STL file of each femur was imported and analyzed using an algorithm designed to calculate automatically the femoral angles on isolated reconstructions of an individual bone (Figure 1C) (15).

**Figure 2:**
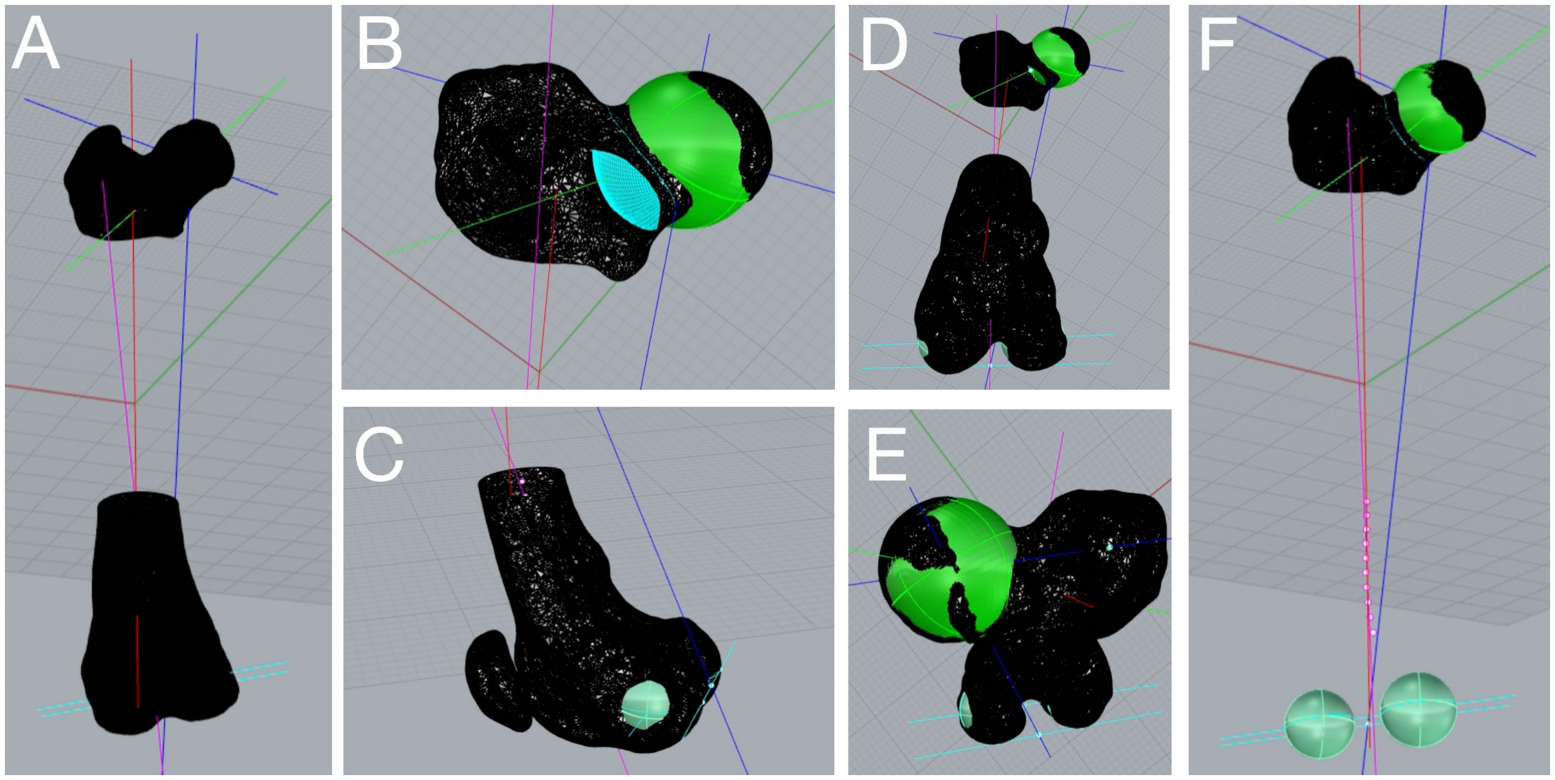
Three-dimensional (3D) computational analysis of a canine femur affected by medial patellar luxation grade 3. After importing the femoral 3D model in computer-aided design software, the 3D automated computation starts by clicking on the femoral head. The anatomical and mechanical axes (multicolored lines) appeared **(A)** along with the angular measurements. The femoral head is superimposed with a best-fitting sphere (green, **B**), and the femoral neck is defined by a section parallel to the femoral head (light blue, **B**). The femoral condyles are superimposed with two best-fitting spheres (light green, **C**), and the distal joint orientation line (light blue, **C**) is found as the tangent axis in the distocaudal point of the condyles. Distoproximal and proximodistal axial views of the analyzed femur **(D,E)**. Images showing the 3D femoral axes and the femoral condyles best-fitting spheres after removal of the distal femur mesh **(E,F)**.

The algorithm was developed to run on a specific commercial CAD software platform (Rhinoceros version 5, Robert McNeel & Associates). Once the STL file was imported into the software, only two manual operations were required to start the 3D automated computation: (1) removal of the internal femoral mesh, if present, and (2) selection of an arbitrary point of the femoral head (Video S1). After these manual procedures, the algorithm analyzed the 3D femoral model in a proximo to distal direction (Figures 1D, 2A) and performs the computation of all anatomical and mechanical femoral angles. For the purpose of this study, the femoral angles measured were the anatomical lateral distal femoral angle (aLDFA), the femoral neck angle (FNA), and the femoral torsion angle (FTA).

Once the 3D computation was performed, these angles were displayed on the top bar of the software and recorded. The operator can assess the quality of the analysis of the femoral head by inspecting the appropriateness of a rendered best-fitting sphere (Figure 2B). The system was developed to include all the mesh vertices belonging to the femoral head, while excluding those belonging to the acetabulum. The area of the femoral neck was found through a curvilinear section that is parallel to the femoral head sphere (Figure 2B). The algorithm calculates the center of the femoral neck and connects it to the center of the femoral head to delineate the femoral head and neck axis. Two best-fitting spheres are superimposed on the femoral condyles (Figures 2C–F). The most distocaudal points of each femoral condyle can be assessed to verify whether the transcondylar axis is accurately drawn (Video S1). In the case where a suboptimal best-fitting sphere of the femoral head or condyles was observed, the user could change manually specific parameters in a dedicated toolbar to improve the quality of the computation.

### Evaluation of the 3D automated femoral angle measurement

The 3D automated femoral measurements were used to (1) evaluate the femoral alignment, (2) decide whether traditional techniques (trochleoplasty, tibial tuberosity transposition, and fascia imbrication) or femoral corrective osteotomies were required to treat PL; (3) used as reference intraoperative values for femoral corrective osteotomies.

**Figure 3:**
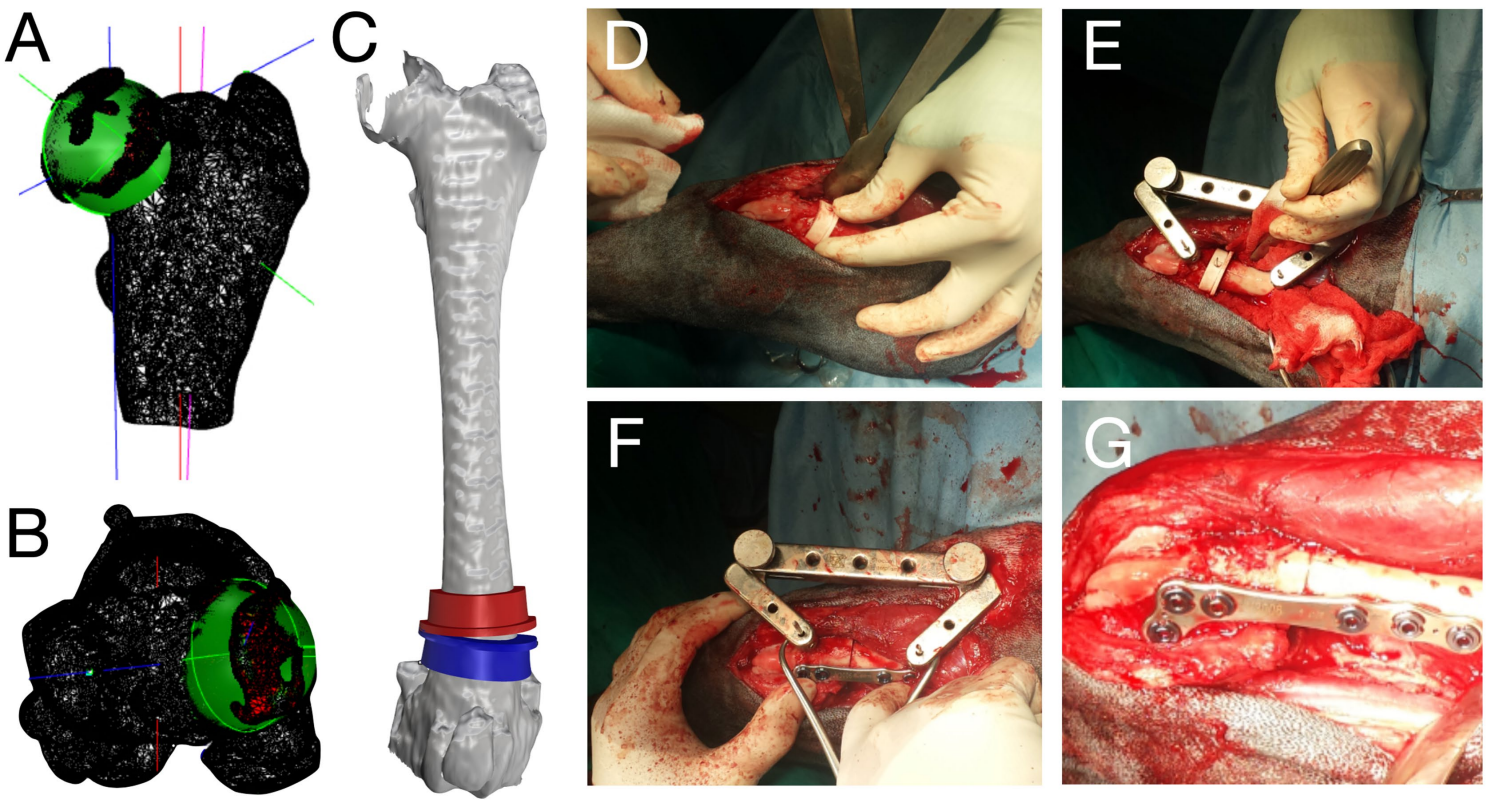
Preoperative images of three-dimensional (3D) computation and intraoperative pictures for the surgical treatment of grade 2 medial patellar luxation. Frontal and axial views of the 3D femoral computation **(A,B)**. Cutting guides on the 3D femoral model to correct femoral varism and retroversion **(C)**. Intraoperative pictures showing the guides placement **(D)**, jig application **(E)**, and osteotomy reduction and plate fixation **(F,G)**.

For instance, in the case of normal or mildly abnormal 3D femoral angles (i.e., 90° < aLDFA <102°) (6, 29) with no other femoral deformities, traditional techniques were performed. Whereas in case of pathologic femoral torsion (FTA < 18° or > 35°) and or pathological frontal malalignment (aLDFA <90° or > 102°), femoral corrective osteotomies were recommended for the surgical plan (Figure 3). Because 3D computation could only be used for the femur, CT was used for tibial measurements and surgical planning. The surgical techniques performed on each case were recorded.

### Postoperative radiographic findings

Because no CT evaluation was performed after surgery, the orthogonal radiographic study was used to assess the hindlimb alignment, the patellar position, and implant positioning. The aLDFA and FNA were measured using previously described methods (6, 24). The FTA was measured using the geometrical biplanar technique (24, 30, 31). The tibial torsion measurement was performed by evaluating, in a straight tibial caudocranial projection, the position of the tibial tuberosity in relation to the pes (5, 24). The tibial frontal alignment was assessed using the methodology described by Dismukes et al. (25). Follow-up radiographs were performed at scheduled intervals that usually were at 1, 2, and 3 months after surgery. Postoperative radiographic findings were evaluated in combination with clinical examination to evaluate the patellar position and thus evaluate the success of the surgical treatment.

### Postoperative clinical findings

The follow-up was classified as referring to literature guidelines (22). Each dog was examined during the follow-up by the surgeon or surgical assistant at scheduled intervals. The orthopedic examination included lameness scoring, evaluation of the surgical wound, and palpation of the operated limb to assess patellar tracking and position. The patellar tracking, lameness score, and complications were used to classify the clinical outcomes as full functional, acceptable functional, and unacceptable functional (5). In detail, full functional was deemed when the patella was *in situ*, the lameness degree was 0/5 or 1/5, and none to minor complications were observed. An acceptable function was defined with the patella *in situ*, the lameness degree was 1/5 or 2/5, and none to major complications were observed. An unacceptable function was deemed whether the patella was *in situ* or luxated, the lameness degree was equal or greater than 3/5, and major complication was observed.

## Results

### Demographic data

The data from 73 hindlimbs were initially collected, and 26 hindlimbs were excluded from the study because no CT was performed. The records of 41 dogs were reviewed, and 47 hindlimbs were included in the study. The signalment, body weight, direction, and degree of PL are shown in Table 1. Mixed-breed dogs were overrepresented (*n* = 13). Median age of the dogs was 7.5 years (range: 0.6–13.4 years). Median body weight was 13.8 kg (range: 2.1–57 kg).

**Table 1:** Demographic data, preoperative lameness, and patellar luxation scores.

	Breed	Age	Body weight (kg)	Lameness degree	Patellar luxation (direction and degree)	Additional diseases
1	Labrador R.	5	28.2	R 3/5	R MPL 3/4	R hip OA R CCLR
2	Mixed-breed	0.8	37.4	L 3/5	L MPL 3/4	L hip OA
3	Boule dog	7	8.4	R 2/5	R MPL 2/4 L MPL 1/4	R hip OA
4	Appenzeller	6.2	19	R 2/5	R MPL 2/4	R Stifle OA
5	Mixed-breed	2.5	8.6	R 2/5	R MPL 2/4 L MPL 1/4	
6	Newfoundland	0.9	41	L 3/5	L LPL 3/4	L hip OA
7	Mixed-breed	2.4	6.2	L 3/5	L MPL 3/4	
8	Mixed-breed	1.4	31	R 3/5	R LPL 3/4	R hip OA
9	Mixed-breed	3.3	5.5	R 2/5	R MPL 2/4	
10	Mixed-breed	3.1	6	R 2/5 L 1/5	R MPL 2/4 L MPL 1/4	
11	Mixed-breed	3.1	4.5	R 1/5 L 1/5	R MPL 2/4 L MPL 1/4	R Stifle OA
12	Mixed-breed	8.5	7.8	L 2/5	L MPL 2/4	
13	Yorkshire T.	4.3	4.5	R 2/5 L 1/5	R MPL 3/4 L MPL 2/4	R hip OA R CCLR
14	Appenzeller	10.3	21	R 2/5	R MPL 3/4	R hip OA R Stifle OA
15	Yorkshire T.	1.1	5.5	R 3/5 L 3/5	R MPL 4/4 L MPL 4/4	
16	Mixed-breed	1.9	7.2	R 3/5 L 1/5	R LPL 4/4 L LPL 2/4	
17	Yorkshire T.	8.7	6.4	R 2/5 L 1/5	R MPL 3/4	
18	Yorkshire T.	11.3	5.3	R 2/5	R MPL 2/4 L MPL 1/4	
19	Mixed-breed	3.1	17	R 3/5	L MPL 4/4	R hip OA
20	Mixed-breed	0.9	4.3	R 3/5 L 3/5	R LPL 4/4 L LPL 4/4	R hip OA L hip OA
21	San Bernard	0.9	47.6	R 2/5	R MPL 2/4	R hip OA
22	Mixed-breed	0.11	3.7	R 3/5 L 3/5	R LPL 4/4 L LPL 4/4	
23	Weimaraner	0.8	17.4	L 3/5	L MPL 3/4	L Stifle OA
24	Maltese	9	4.5	R 2/5	R MPL 4/4	
25	Chihuahua	3.2	2.4	L 3/5	L MPL 3/4	
26	Shiba-Inu	4.7	9	R 2/5	R MPL 3/4	
27	Maltese	6.4	3.2	R 2/5	R MPL 3/4	R CCLR
28	Boule dog	4.2	9.8	R 2/5	R MPL 2/4	
29	American Staffordshire	3.3	24.7	R 2/5	R MPL 2/4	R CCLR R Stifle OA
30	Chihuahua	1.5	2	R 1/5 L 1/5	R MPL 2/4 L MPL 2/4	
31	Labrador R.	2.5	28	R 2/5	R MPL 2/4	R hip OA R CCLR
32	Segugio Italiano	7.2	16.8	R 4/5	R MPL 4/4	R hip OA R Stifle OA
33	Setter inglese	3.3	17.8	L 2/5	L LPL 3/4	
34	Bolognese	1.9	5.5	R 3/5 L 3/5	R MPL 4/4 L MPL 2/4	
35	Yorkshire	1.7	2.1	R 4/5	R MPL 4/4	
36	Segugio Italiano	4.6	17.2	R 2/5	R MPL 3/4	R hip OA
37	Labrador R.	7.8	26.4	R 1/5	R MPL 2/4	
38	Chihuahua	2.1	2.7	R 1/5 L 1/5	R MPL 2/4 L MPL 2/4	
39	Labrador R.	7.8	27	R 1/5	R MPL 4/4	R hip OA R Stifle OA
40	Yorkshire	6.4	4.7	R 1/5 L 1/5	R MPL 2/4 L MPL 2/4	
41	Mixed-breed	5.2	26.5	R 2/5	R MPL 2/4	

Right (R); left (L), medial patellar luxation (MPL), lateral patellar luxation (LPL), osteoarthritis (OA), cranial cruciate ligament rupture (CCLR).

### Preoperative clinical findings

Grade 2/5 (40%) was the most observed lameness score (19/47), followed by grade 3/5 (31%, 15/47, Table 1). Medial patellar luxation (MPL) was found in 81% of the dogs (38/47), while lateral patellar luxation (LPL) was diagnosed in 19% of cases (9/47). The majority (46%) of dogs presented with grade 3 PL (22/47), followed by grade 2 PL in 33% (18/47 limbs, Table 1). Hip dysplasia (23%, 11/47) and a cranial cruciate ligament rupture (17%, 8/47) were the most common additional diseases observed (Table 1).

### Preoperative 3D femoral angle measurements

Mean (±SD, range) aLDFA, FNA, and FTA in dogs affected by MPL were 101.4° (±3.6, 89°–114°), 132.5° (±2.6, 117°–141°), 17.6° (±4.3, 8°–26°), respectively. The mean (±SD, range) aLDFA, FNA, and FTA in dogs affected by LPL were 89.3° (±7.6, 47°–94°), 134.8° (±2.9, 119°–143°), and 36.9° (±5.3, 25°–50°), respectively.

In case where corrective osteotomies were performed based on the preoperative 3D measurements, the mean (±SD, range) aLDFA, FNA, and FTA were 104.5° (±4.5, 99°–114°), 133.7° (±2.8, 124°–141°), and 12.9° (±2.7, 8°–18°) respectively, in MPL-affected dogs, whereas, in LPL-affected dogs, the mean (±SD, range) aLDFA, FNA, and FTA were 87.2° (±13.1, 47°–91°), 135.7° (±2.7, 123°–138°), and 41.4° (±7, 33°–50°), respectively. In the case where traditional techniques or non-conventional techniques were performed, the mean (±SD, range) aLDFA, FNA, and FTA were 98.3° (±2.7, 89°–99°), 133.3° (±2.4, 120°–141°), and 22.4° (±5.9, 18°–26°), respectively, in MPL-affected dogs, whereas, in LPL-affected dogs, the mean (±SD, range) aLDFA, FNA, and FTA were 91.4° (±2.2, 90°–94°), 134° (±3.1, 125°–138°), and 32.4° (±3.7, 22°–36°), respectively.

### Intraoperative data

The traditional techniques to treat PL included the following: trochleoplasty (wedge or block resection), fascia imbrication with or without contralateral fascia release, and tibial tuberosity transposition (TTT). The corrective osteotomies included distal femoral osteotomy (DFO), femoral detorsional osteotomy (FDO), tibial detorsional osteotomy (TDO), and proximal tibial osteotomy (PTO). The non-conventional surgical techniques included the following: patellar groove replacement (PGR), arthroscopically assisted fascia plication, and trochlear ridge prosthesis (TRP) (32).

Corrective osteotomies were performed in 72% of cases (34/47, Figure 3): distal femoral osteotomy (DFO, *n* = 25), femoral detorsional osteotomy (FDO, *n* = 18), proximal tibial medial osteotomy (PTO, *n* = 2), and tibial detorsional osteotomy (TDO, *n* = 12).

At least one traditional technique was performed in 80% of cases (38/47): fascia imbrication (*n* = 38), wedge trochleoplasty (*n* = 10), bloc trochleoplasty (*n* = 6), fascia and joint capsule release (*n* = 14), and tibial tuberosity transposition (*n* = 20). Non-conventional techniques were performed in 23% of cases (11/47): custom-made trochlear ridge prosthesis (*n* = 7), arthroscopically assisted capsular plication (*n* = 3), and PGR (*n* = 1).

### Postoperative radiographic findings

The radiographic follow-up of this study was defined as short term as the median for radiographic recheck was 4.7 months (range: 2 to 27 months, Table 2). The median time for bone healing was 2.8 months (range: 2–4 months). The postoperative mean (±SD, range) aLDFA, was 94.6° (±2.1, range: 91.7°–97.4°). With regard to the radiographic axial femoral, the mean FTA was 25.7° (±3.3, range: 23.1°–32.8°). When TDO was performed, the tibial tuberosity was found in a central position in 9 of 11 cases (Table 2). The two medial PTO were successful in restoring the physiological frontal tibial alignment as the mean (±SD, range) postoperative mMPTA was 93.1° (±1.4, 91.3°–95°).

**Table 2:** Intraoperative and postoperative descriptive data of radiographic and clinical outcomes after PL luxation treatment based on 3D automated femoral planning.

Cases	Unilateral vs. bilateral	Surgeries	Radiographic follow-up	Postoperative patellar position	Postoperative lameness score	Clinical outcomes	Complications
1	Unilateral	DFO TTT TPLO	2	*In situ*	0/5	Full functional	
2	Unilateral	DFO FDO	2	*In situ*	0/5	Full functional	
3	Unilateral	DFO	2	*In situ*	0/5	Full functional	
4	Unilateral	Arthroscopic CP TTT	2	*In situ*	0/5	Full functional	
5	Unilateral	Block trochleoplasty TTT	7	*In situ*	0/5	Full functional	
6	Unilateral	Block Trochleoplasty TTT Revision: DFO, FDO	23	LPL Revision: *In situ*	1/5	Acc. functional	Patellar luxation Delayed bone union
7	Unilateral	DFO TRP TTT	13	*In situ*	0/5	Full functional	
8	Unilateral	DFO FDO	5	*In situ*	0/5	Full functional	
9	Unilateral	Wedge Trochleoplasty TTT	6	*In situ*	0/5	Full functional	Periarticular fibrosis
10	Unilateral	Wedge Trochleoplasty TTT	4	*In situ*	0/5	Full functional	
11	Unilateral	Arthroscopic CP TTT	6	MPL	2/5	Acc. functional	Patellar luxation
12	Unilateral	TRP TTT	9	*In situ*	0/5	Full functional	
13	Unilateral	DFO FDO TDO	2	*In situ*	0/5	Full functional	
14	Unilateral	DFO Wedge Trochleoplasty TTT	13	*In situ*	1/5	Acc. functional	Kirschner wire migration
15 R	bilateral	DFO TRP TDO	2	*In situ*	0/5	Full functional	
15 L		DFO TRP TDO	2	*In situ*	0/5	Full functional	
16	Unilateral	DFO TTT Block Trochleoplasty	10	*In situ*	1/5	Acc. functional	Seroma Screw fracture
17	Unilateral	TRP TTT	2	*In situ*	0/5	Full functional	
18	Unilateral	Block Trochleoplasty TTT	2	*In situ*	0/5	Full functional	
19	Unilateral	DFO FDO Wedge Trochleoplasty TTT	2	*In situ*	0/5	Full functional	
20 R	Bilateral	Wedge Trochleoplasty TTT Revision: DFO, FDO	2	MPL	1/5	Acc. functional	Patellar luxation
20 L		DFO FDO	2	*In situ*	0/5	Full functional	
21	Unilateral	DFO FDO	2	*In situ*	0/5	Full functional	
22 R	Bilateral	DFO FDO TRP	2	*In situ*	0/5	Full functional	
22 L		DFO FDO TRP	2	*In situ*	0/5	Full functional	
23	Unilateral	DFO FDO TDO	2	*In situ*	0/5	Full functional	
24	Unilateral	DFO TDO	12	*In situ*	0/5	Full functional	
25	Unilateral	Block Trochleoplasty TTT	2	MPL	2/5	Unacceptable functional	Patellar luxation
26	Unilateral	DFO FDO TDO	2	*In situ*	0/5	Full functional	
27	Unilateral	Block Trochleoplasty FDO TPLO	3	*In situ*	1/5	Acc. functional	Delayed bone union
28	Unilateral l	DFO TTT	2	*In situ*	0/5	Full functional	
29	Unilateral	FDO PGR TPLO	2	*In situ*	0/5	Full functional	Kirschner wire migration
30 R	Bilateral	Block Trochleoplasty TDO	6	*In situ*	0/5	Full functional	
30 L		Block Trochleoplasty TDO	3	*In situ*	0/5	Full functional	
31	Unilateral	DFO TPLO	2	*In situ*	0/5	Full functional	
32	Unilateral	DFO FDO TDO	2	*In situ*	0/5	Acc. functional	Seroma
33	Unilateral	FDO TTT	4	*In situ*	0/5	Full functional	
34	Unilateral	Block Trochleoplasty DFO FDO TDO	3	*In situ*	0/5	Acc. functional	Patellar tendon tendinitis
35	Unilateral	DFO Block Trochleoplasty	2	MPL	4/5	Unacceptable functional	Patellar luxation
36	Unilateral	Block Trochleoplasty FDO PTO	4	*In situ*	0/5	Full functional	Kirschner wire migration
37	Unilateral	DFO PTO	2	*In situ*	0/5	Full functional	
38 R	Bilateral	Wedge Trochleoplasty TDO	2	*In situ*	0/5	Full functional	
38 L		TDO					
39	Unilateral	Block Trochleoplasty DFO FDO TDO		*In situ*	1/5	Acc. functional	
40 R	Bilateral	TTT		*In situ*	0/5	Full functional	Incisional seroma
40 L		TTT					
41	Unilateral	Arthroscopic CP		*In situ*	0/5	Full functional	

Distal femoral osteotomy (DFO), femoral detorsional osteotomy (FDO), tibial detorsional osteotomy (TDO), proximal tibial osteotomy (PTO), tibial tuberosity transposition (TTT), trochlear ridge prosthesis (TRP), patellar groove replacement (PGR), tibial plateau leveling osteotomy (TPLO), and capsular plication (CP).

### Postoperative clinical findings

Overall, PL was successfully treated using preoperative 3D femoral angles in 96% of the cases (45/47). In these cases, at the time of the last clinical recheck, the patella was tracking properly within the femoral trochlear groove during stifle range of motion execution. As concerns PL scoring, there was an improvement in almost all the dogs treated 98% (46/47). The majority of them 96% (45/47) were scored as grade 0, while two cases were scored as grade 3 MPL. Specifically, only one case did not improve the PL score, as it was rated both pre- and postoperatively as grade 3 MPL. All the grade 4 PL (*n* = 7) improved postoperatively: grade 0 PL (*n* = 5), grade 1 PL (*n* = 1), and grade 3 PL (*n* = 1).

The preoperative lameness score improved in 98% of the cases (46/47) at final clinical follow-up (Table 2). The majority of the cases 78% (37/47) were scored postoperatively as grade 0/5. Only one case was scored pre- and postoperatively as a grade 4/5. Considering postoperative femoral alignment, patellar tracking, lameness and PL score, and complications observed, 72% of the cases (34/47) had fully functional clinical outcomes, 23% (11/47) had an acceptable functional clinical outcome, and 4% (2/47) had an unacceptable clinical outcome (Table 2). All dogs with a full functional outcome showed no visible lameness during the last clinical recheck, while two dogs with grade 0/5 postoperative lameness were deemed as acceptable function as they were affected by minor complications. The other eight cases scored as acceptable function showed grade 1/5 postoperative lameness and had some minor complications (Table 2).

### Complications

A major complication was recorded in 10% of the operated limbs (5/47) and a minor complication was observed in 25*%* (11/47) of the limbs. Recurrent patella luxation was the only major complication observed and occurred in five of five limbs with major complications. In three of five cases, the surgeon performing the surgery did not include the results of the 3D aCAD analysis in their surgical plan, meaning that traditional techniques were performed instead of femoral corrective osteotomies. The three cases were revised performing DFO (*n* = 2), FDO (*n* = 1), and TDO (*n* = 1). Patellar luxation was successfully treated in all three cases. In the last two cases, the 3D planning failed to provide the correct surgical planning. One case was grade 3 MPL and one grade 4 MPL in two small breed dogs. The grade 4 improved to grade 3 MPL, while the PL score in the grade 3 MPL remained unchanged. In both cases, owner refused further surgical treatment. Minor complications included the following: k-wire migration necessitating removal (*n* = 3), seroma (*n* = 2), delayed bone union (*n* = 2), painful periarticular fibrosis (*n* = 1), incisional self-limiting seroma (*n* = 1), screw fracture not requiring revision (*n* = 1), patellar tendon tendinitis (*n* = 1).

## Discussion

In this study, we measured three femoral angles in dogs affected by PL using a 3D automated methodology. The 3D femoral angles were used as measurements during the preoperative planning and surgical treatment of PL. We accept our hypothesis as PL was successfully treated in >95% of the cases. Furthermore, 45 of 47 of the cases showed a full or acceptable functional outcome at the last clinical recheck. At the final clinical follow-up, both lameness and PL score improved in 46 of 47 of the cases. Patellar reluxation occurred in five dogs. However, in three of five cases the 3D preoperative plan was not followed by the designated surgeon.

The overall complication rate of this study was 35%, including mainly minor complications (25%). All the major complications observed (10%) were due to PL recurrence. When comparing this study with similar publications on PL (12, 33), our complication rate is lower in terms of major complications, and similar or lower when considering the overall complication rate (10, 12). In three cases of PL recurrence, the preoperative surgical plan was not attended by the surgeon, who opted initially for traditional techniques instead of corrective osteotomies, as suggested by the 3D femoral measurements. However, in two cases, the 3D measurements failed to provide the correct preoperative reference values, and thus, the surgical execution failed to treat the PL. Both cases were toy breed dogs with either grade 3 or 4, and they were affected by stifle and hip osteoarthritic changes. Possible explanations for the incorrect measurements may rely on the relatively small size of the femur and the abnormally altered shape of the femoral condyles. It could be also possible that the surgical execution was the main reason for the surgical failure, but we cannot prove this statement. In toy breed dogs, a 1-mm discrepancy for a femoral closing wedge osteotomy or detorsional osteotomy can create a great mismatch between preoperative and postoperative measurements. As a result, an under- or overcorrection of the bone deformity is achieved. Most likely both cases may have benefited from 3D-printed cutting guides for the correction of femoral and tibial deformities.

In this study, we included cases affected by hip dysplasia and cranial cruciate ligament rupture. Dogs with PL may be often affected by moderate-to-severe osteoarthritis of the hip and stifle joints. All these conditions lead to morphological changes in the joint that may create challenges for the quality of the 3D femoral computation. These joint abnormalities are frequently observed in grade 3 and grade 4 PL. In this study, 29 cases of PL grade 3 or 4 were analyzed and treated. Specifically, we included seven cases of grade 4 PL, which are the most challenging cases to assess, as they are usually characterized by multiplanar deformities and other concurrent orthopedic diseases such as femoral trochlea dysplasia. In five cases, PL was successfully treated, while one had a postoperative grade 3 MPL and one grade 1 MPL, which did not require surgical revision. Six out of seven dogs had either an acceptable or full function outcome. Even though the group of grade 4 of PL is small, our success rate is in agreement with Dunlap et al. (12) who reported an identical rate of patellar reluxation, while we had a worse outcome compared to Brower et al. (10), as the authors reported no patellar recurrence in 14 stifles affected by grade 4 PL.

The mean preoperative aLDFA and FTA, when a femoral corrective osteotomy was performed, were > than 102° and < than 15, respectively, in case of MPL, and < 88° and the FTA > 40° with LPL. These cutoff values are usually adopted to decide whether a femoral corrective osteotomy or a traditional technique is needed (7, 11). The mean postoperative aLDFA and FTA were 94.6° and 25.7°, respectively. These values fall within the range of a femoral physiological alignment (6–8, 24). Although the reference values used in this study (6, 8, 24) were obtained by using either radiography or CT, we can speculate that the 3D measurements may be a reliable methodology as well. Previous publications showed that the 3D automated femoral measurement had a superior precision (21) than CT and radiographic techniques and was also highly accurate (14) to measure femoral angles in either healthy or pathologic femurs. Additionally, recent studies using a different 3D methodology (16, 17) showed that the 3D measurements can be feasibly performed in both the canine femur and tibia. The use of automated measurements can avoid or, at least decrease, the frequency of image-related errors, thus simplifying the measurements as well as reducing the range of potential manual errors (6). Furthermore, the manual identification of challenging target anatomical landmarks, such as the femoral neck width, is no longer necessary.

This study has some limitations. It is a retrospective study, and thus some of the medical and surgical records may be inaccurate. Due to financial cost for the owners, it was not possible to perform in most of the cases a CT scan after surgery, and thus, no postoperative 3D computation was done. We assessed the postoperative alignment based on radiographs. Femoral torsion was assessed by using a previously described and validated biplanar geometrical approach (5, 24). Tibial torsion was assessed by evaluating the tibial tuberosity position in a straight caudocranial tibial radiographic projection. No objective clinical postoperative measurements such as force plate analysis or validated owner questionnaire have been performed. Therefore, all the clinical postoperative measurements were based on the subjective preoperative and postoperative evaluation of the surgeon.

To the best of the authors’ knowledge, this is the first clinical study reporting surgical data after using a 3D automated femoral measurement methodology in dogs affected by PL. In 96% of the cases, the 3D automated femoral measurements led to a successful surgical correction of PL. Therefore, we conclude that the 3D automated femoral computation is a reliable methodology to be used in clinical cases affected by PL. We also observed that 3D femoral computation can be reliably used for measurements in femurs affected by osteoarthritis of the hip and stifle. The main benefits of using such 3D technology mainly rely on the decrease in all the manual operations and operator-related biases that are commonly related to manual femoral angle measurement. Further developments and future studies on 3D tibial computation are needed to complement this methodology and allow for a thorough 3D analysis of the hindlimb in case of PL.

## Data Availability

The original contributions presented in the study are included in the article/Supplementary material, further inquiries can be directed to the corresponding author.

## Supplementary material

Video S1 Three-dimensional automated computational process for the measurement of the femoral angles in dogs.

## References

[1] Alam R Lee JL Kang HS Kim LS Park SY Lee KC. Frequency and distribution of patellar luxation in dogs 134 cases (2000 to 2005). Vet Comp Orthop Traumatol. (2007) 20:59–64. doi: 10.1055/s-0037-1616589, PMID: 17364098

[2] Bound N Zakai D Butterworth SJ Pead M. The prevalence of canine patellar luxation in three centres. Vet Comp Orthop Traumatol. (2017) 22:32–7. doi: 10.3415/VCOT-08-01-0009 19151868

[3] Perry KL Dejardin LM. Canine medial patellar luxation. J Small Anim Pract. (2021) 62:315–35. doi: 10.1111/jsap.13311 33600015

[4] DeCamp CE Johnston SA Déjardin LCM Schaefer SL. Brinker, piermattei and Flo’s handbook of small animal orthopedics and fracture repair. st Louis, USA: Elsevier (2016).

[5] Longo F Nicetto T Knell SC Evans RB Isola M Pozzi A. Three-dimensional volume rendering planning, surgical treatment, and clinical outcomes for femoral and tibial detorsional osteotomies in dogs. Vet Surg. (2022) 51:1126–41. doi: 10.1111/vsu.13882, PMID: 36054415 PMC9805109

[6] Tomlinson J Fox D Cook JL Keller GG. Measurement of femoral angles in four dog breeds. Vet Surg. (2007) 36:593–8. doi: 10.1111/j.1532-950X.2007.00309.x, PMID: 17686134

[7] Palmer RHIC Cadmus JM. Comparison of femoral angulation measurement between radiographs and anatomic specimens across a broad range of Varus conformations. Vet Surg. (2011) 40:1023–8. doi: 10.1111/j.1532-950X.2011.00895.x, PMID: 22319778

[8] Dudley RM Kowaleski MP Drost WT Dyce J. Radiographic and computed tomographic determination of femoral Varus and torsion in the dog. Vet Radiol Ultrasound. (2006) 47:546–52. doi: 10.1111/j.1740-8261.2006.00184.x, PMID: 17153063

[9] Peterson JL Torres BT Hutcheson KD Fox DB. Radiographic determination of normal canine femoral alignment in the sagittal plane: a cadaveric pilot study. Vet Surg. (2020) 49:1230–8. doi: 10.1111/vsu.13465, PMID: 32484579

[10] Brower BE Kowaleski MP Peruski AM Pozzi A Dyce J Johnson K. Distal femoral lateral closing wedge osteotomy as a component of comprehensive treatment of medial patellar luxation and distal femoral varus in dogs. Vet Comp Orthop Traumatol. (2017) 30:20–7. doi: 10.3415/VCOT-16-07-0103, PMID: 27935008

[11] DeTora MD Boudrieau RJ. Complex angular and torsional deformities (distal femoral malunions). Preoperative planning using stereolithography and surgical correction with locking plate fixation in four dogs. Vet Comp Orthop Traumatol. (2016) 29:416–25. doi: 10.3415/VCOT-15-08-0145, PMID: 27439728

[12] Dunlap AE Kim SE Lewis DD Christopher SA Pozzi A. Outcomes and complications following surgical correction of grade IV medial patellar luxation in dogs: 24 cases (2008-2014). J Am Vet Med Assoc. (2016) 249:208–13. doi: 10.2460/javma.249.2.208, PMID: 27379597

[13] Paley D Herzenberg JE, Eds. Principles of deformity correction. Berlin: Springer-Verlag. (2005). 703–710.

[14] Longo F Savio G Contiero B Meneghello R Concheri G Franchini F. Accuracy of an automated three-dimensional technique for the computation of femoral angles in dogs. Vet Rec. (2019) 185:443. doi: 10.1136/vr.105326, PMID: 31292274

[15] Savio G Baroni T Concheri G Baroni E Meneghello R Longo F. Computation of femoral canine morphometric parameters in three-dimensional geometrical models. Vet Surg. (2016) 45:987–95. doi: 10.1111/vsu.12550, PMID: 27716955

[16] Brühschwein A Schmitz B Zöllner M Reese S Meyer-Lindenberg A. Introduction of a bone-centered three-dimensional coordinate system enables computed tomographic canine femoral angle measurements independent of positioning. Front Vet Sci. (2022) 9:1019215. doi: 10.3389/fvets.2022.1019215, PMID: 36504862 PMC9730830

[17] Brühschwein A Schmitz B Zöllner M Reese S Meyer-Lindenberg A. Computed tomographic angular measurements using a bone-centered three-dimensional coordinate system are accurate in a femoral torsional deformity model and precise in clinical canine patients. Front Vet Sci. (2023) 10:1019216. doi: 10.3389/fvets.2023.1019216, PMID: 37138905 PMC10149667

[18] Longo F Penelas A Gutbrod A Pozzi A. Three-dimensional computer-assisted corrective osteotomy with a patient-specific surgical guide for an antebrachial limb deformity in two dogs. SAT. (2019) 161:473–9. doi: 10.17236/sat00216, PMID: 31298216

[19] Worth AJ Crosse KR Kersley A. Computer-assisted surgery using 3D printed saw guides for acute correction of antebrachial angular limb deformities in dogs. Vet Comp Orthop Traumatol. (2019) 32:241–9. doi: 10.1055/s-0039-1678701 30965369

[20] De Armond CC Lewis DD Kim SE Biedrzycki AH. Accuracy of virtual surgical planning and custom three-dimensionally printed osteotomy and reduction guides for acute uni- and biapical correction of antebrachial deformities in dogs. J Am Vet Med Assoc. (2022) 260:1–9. doi: 10.2460/javma.21.09.0419, PMID: 35460550

[21] Cook JL Evans R Conzemius MG Lascelles BDX McIlwraith CW Pozzi A. Proposed definitions and criteria for reporting time frame, outcome, and complications for clinical orthopedic studies in veterinary medicine. Vet Surg. (2010) 39:905–8. doi: 10.1111/j.1532-950X.2010.00763.x, PMID: 21133952

[22] Aper R Kowaleski MP Apelt D Drost WT Dyce J. Computed tomographic determination of tibial torsion in the dog. Vet Radiol Ultrasound. (2005) 46:187–91. doi: 10.1111/j.1740-8261.2005.00048.x, PMID: 16050274

[23] Petazzoni M Jaeger GH: Atlas of clinical goniometry and radiographic measurements of the canine pelvic limb. Merial. (2008).

[24] Dismukes DI Tomlinson JL Fox DB Cook JL Song KJ. Radiographic measurement of the proximal and distal mechanical joint angles in the canine tibia. Vet Surg. (2007) 36:699–704. doi: 10.1111/j.1532-950X.2007.00323.x, PMID: 17894597

[25] Putnam RW. Patellar luxation in the dog University of Guelph. (master thesis) (1968).

[26] Nicetto T Longo F. Trochlear ridge prostheses for reshaping femoral trochlear ridges in dogs with patellar luxation. Vet Comp Orthop Traumatol. (2023) 37:098–106. doi: 10.1055/s-0043-1776331 PMC10932614 37907244

[27] Nicetto T Longo F Contiero B Isola M Petazzoni M. Computed tomographic localization of the deepest portion of the femoral trochlear groove in healthy dogs. Vet Surg. (2020) 49:1246–54. doi: 10.1111/vsu.13426, PMID: 32343440

[28] Longo F Nicetto T Banzato T Savio G Drigo M Meneghello R. Automated computation of femoral angles in dogs from three-dimensional computed tomography reconstructions: comparison with manual techniques. Vet J. (2018) 232:6–12. doi: 10.1016/j.tvjl.2017.11.014, PMID: 29428094

[29] Swiderski JK Radecki SV Park RD Palmer RH. Comparison of radiographic and anatomic femoral varus angle measurements in normal dogs. Vet Surg. (2008) 37:43–8. doi: 10.1111/j.1532-950X.2007.00347.x, PMID: 18199056

[30] Bardet J Rudy R Hohn R. Measurement of femoral torsion in dogs using a biplanar method. Vet Surg. (1983) 12:1–6. doi: 10.1111/j.1532-950X.1983.tb00693.x

[31] Montavon P Hohn R Olmstead M Rudy R. Inclination and anteversion angles of the femoral head and neck in the dog evaluation of a standard method of measurement. Vet Surg. (1985) 14:277–82. doi: 10.1111/j.1532-950X.1985.tb00883.x

[32] Longo F Memarian P Knell SC Contiero B Pozzi A. Computed tomographic measurements of the femoral trochlea in dogs with and without medial patellar luxation. Vet Surg. (2023) 52:395–406. doi: 10.1111/vsu.13903, PMID: 36196803

[33] Arthurs GI Langley-Hobbs SJ. Complications associated with corrective surgery for patellar luxation in 109 dogs. Vet Surg. (2006) 35:559–66. doi: 10.1111/j.1532-950X.2006.00189.x, PMID: 16911156

